# Impact of continuing education on maternal and child health indicators

**DOI:** 10.1371/journal.pone.0235258

**Published:** 2020-06-26

**Authors:** Débora Dupas Gonçalves do Nascimento, Sílvia Helena Mendonça de Moraes, Carlos Antonio de Souza Teles Santos, Albert Schiaveto de Souza, Rafael Aiello Bomfim, Alessandro Diogo De Carli, Vera Lucia Kodjaoglanian, Mara Lisiane de Moraes dos Santos, Edilson José Zafalon

**Affiliations:** 1 Oswaldo Cruz Foundation, Campo Grande, Mato Grosso do Sul, Brazil; 2 Gonçalo Muniz Institute, Oswaldo Cruz Foundation, Salvador, Bahia, Brazil; 3 Biosciences Institute, Federal University of Mato Grosso do Sul, Campo Grande, Mato Grosso do Sul, Brazil; 4 Post-doctoral Researcher at The School of Public Health, University of São Paulo, São Paulo, Brazil; 5 Faculty of Dentistry, Federal University of Mato Grosso do Sul, Campo Grande, Mato Grosso do Sul, Brazil; 6 State Secretary of Health, Campo Grande, Mato Grosso do Sul, Brazil; 7 Integrated Health Institute, Federal University of Mato Grosso do Sul, Campo Grande, Mato Grosso do Sul, Brazil; Florida State University College of Medicine, UNITED STATES

## Abstract

**Objective:**

This study investigated whether the presence of care workers who completed a specialization course on family health was associated with improved care and maternal and child health indicators in municipalities in the state of Mato Grosso do Sul, Brazil.

**Methods:**

Negative binomial regression models with fixed effects were used for the 79 municipalities in the state of Mato Grosso do Sul, with repeated observations for the period 2009–2015. For our reference, the parameter “number of professionals who completed the course” calculated the proportion of professionals who completed the course, and was divided by the total number of primary health care professionals in the municipality to create a ratio. The cutoff points used represented tertile distribution: T3: high (0.35–1.00), T2: intermediate (0.02–0.33) and T1: low (0.00–0.01); to avoid biased results, the analysis was also performed for the years prior to the beginning of the course in question (2009 and 2010).

**Results:**

During the study period, enrollment of pregnant women, exclusive breastfeeding for children under 4 months, and up-to-date vaccinations in children younger than 1 year to 23 months increased (high to intermediate categories) in municipalities where professionals who completed the specialization course worked. Growth in the intermediate ratio was also observed in indicators related to cervical cancer screening and new diagnoses of congenital syphilis in infants under one year of age.

**Conclusions:**

The presence of care workers who completed a specialization course on family health was seen to be associated with improved care and indicators for maternal and child health in municipalities in the state of Mato Grosso do Sul, Brazil. These findings reaffirm the importance and effectiveness of policies on training and continuing education for the Brazilian Unified Health System.

## Introduction

In Brazil, the 1988 Constitution established health as a right for all and a responsibility of the government, which created the Unified Health System (SUS). The SUS provides the entire population access to health care and services based on the principles of universality, comprehensiveness, and equity. In order to meet public and individual health needs, especially for low-income populations, the Family Health Strategy (FHS) is at the center of government actions and investments to reorient the health care model (from a hospital-based and individual-focus model to one centered around the family and the community) and implement primary health care (PHC) [1]. The FHS teams are comprised of a general practitioner physician, a nurse, a nursing technician, and community health agents (in some municipalities, they also include a dentist and oral hygienist) and generally work to promote health and prevent disease and care for chronic illnesses and maternal and child health [2].

Since the FHS was established in 1994, the number of teams has grown significantly: in 1998 there were 2,000 teams serving 7 million people (4% of the Brazilian population) [3], while in 2018 this number grew to 43,000 teams with over 700,000 staff responsible for the health of 134 million people (64.7% of the population) [4].

Strong evidence of the impact the FHS has had on important health indicators is associated with this significant increase in the population it covers [5,6]. But despite the growth in team numbers and expansion in the activities and services offered, Brazil faces problems implementing the care model and providing skilled health care. While public access to primary care services has grown undeniably, the quality and effectiveness of the services offered still leave room for improvement [7].

Training for healthcare professionals is one of the initiatives for to improve the FHS, since there is a disconnect between health training and the principles of the SUS [8]. To address this issue, the Brazilian Ministry of Health instituted the National Continuing Education Policy to transform educational practices related to care, management, and organization of work in health through a training process that analyzes routine work experiences [9]. In order to expand and catalyze permanent education activities and provide skilled labor for the SUS, in 2010 the Ministry of Health created the SUS Open University (UNA-SUS) [10].

Within this context, the Oswaldo Cruz Foundation-Mato Grosso do Sul, the Federal University of Mato Grosso do Sul, and UNA-SUS collectively established a specialization course in basic family health care (CEABSF), offered via distance education. This 405-hour course targets public health professionals with higher education who work in the FHS in the state of Mato Grosso do Sul (MS), which is located in the Midwest region of the country. This state was the first in Brazil to train specialists in family health through UNA-SUS in 100% of its municipalities.

The CEABSF course integrated service teaching and continuing education through units on topics such as public health policies, planning, the work process in the FHS, health education, clinical expansion, and health care during different life cycles, including maternal and child health. All of these units were developed from the viewpoint of health promotion and disease prevention, using questioning methodologies that encourage reflection and peer discussion which allow students to use different strategies to (re)organize their work processes.

Studies have demonstrated the effectiveness of online continuing education in improving professional skills in PHC [11,12]. Providing specialist training to professionals across the state through the CEABSF course was expected to produce better health indicators through access to skilled care. In the literature on this topic, we found two studies that showed positive impacts on health indicators from professionals who were trained to work in family health, related to hospitalizations for motives that are sensitive to primary care [1,13] and registering and monitoring patients with diabetes and high blood pressure [1]. Investigations that advance understanding of the relationship between training health professionals and positive impacts on health indicators will support the formulation of new policies on training professionals for PHC, in turn contributing to more effective services [1,13].

The objective of this study was to verify whether the presence of CEABSF graduates in FHS teams was associated with improved care and indicators of maternal and child health in municipalities in the state of Mato Grosso do Sul.

## Methods

This study utilizes a mixed ecological design with a temporal trend, with longitudinal data sets created for the years 2009 to 2015; the units analyzed were the 79 municipalities in the state of Mato Grosso do Sul, Brazil.

The study considered coverage provided by professionals who completed the family health specialization course and served the population in PHC, with a cumulative ratio over the period studied. The coverage of these professionals was established as the exposure variable to verify improvements in PHC indicators, and the model was adjusted for fixed indexes (HDI and Gini coefficient). This adjustment allowed us to determine the effects on the PHC indicators in relation to the proportion of professionals who completed the course. The data for the HDI and Gini coefficient (2012) referring a single year were seen to be sufficient to infer any effects on the PHC indicators in relation to the proportion of professionals who completed the course.

In this study we used a “municipality ratio” to define the proportion of professionals who completed the course among PHC professionals in a given municipality. This ratio was calculated by dividing the total number of professionals who completed the specialization course by the total number of PHC professionals in each municipality in Mato Grosso do Sul. The ratio was then classified by tertiles: T3, high (0.35–1.00); T2, intermediate (0.02–0.33); T1, low (0.00–0.01). We also conducted the same analysis for the years before the specialization course was offered (2009 and 2010) to avoid biased results.

In order to compare and validate our analysis, we also calculated the effect of potential externalities (unrelated to the object of study) in order to exclude positive effects on non-eligible inhabitants living in the municipality with coverage by professionals who completed the course. To do so, we selected data from workplace accidents, cancer-related deaths, and deaths from traffic accidents as control indicators (Table 1).

**Table 1:** Maternal and child health indicators associated with the proportion of professionals who completed the course (“municipality ratio,” Mato Grosso do Sul State, 2009–2015).

	RR	CI (95%)
**Workplace accidents registered**		
**T**_**1(low)**_	1.00	
**T**_**2(intermediate)**_	1.13	0.94–1.36
**T**_**3(high)**_	1.12	0.87–1.43
**Number of cancer-related deaths**		
**T**_**1(low)**_	1.00	
**T**_**2(intermediate)**_	0.96	0.89–1.02
**T**_**3(high)**_	1.01	0.92–1.12
**Number of deaths related to traffic accidents**		
**T**_**1(low)**_	1.00	
**T**_**2(intermediate)**_	1.07	0.95–1.22
**T**_**3(high)**_	1.01	0.85–1.21
**Child development screening programs**		
**T**_**1(low)**_	1.00	
**T**_**2(intermediate)**_	0.96	0.82–1.11
**T**_**3(high)**_	1.20	0.97–1.49
**Prenatal care**		
**T**_**1(low)**_	1.00	
**T**_**2(intermediate)**_	1.00	0.90–1.10
**T**_**3(high)**_	1.02	0.88–1.18
**Cervical cancer screenings**		
**T**_**1(low)**_	1.00	
**T**_**2(intermediate)**_	1.19	1.08–1.31
**T**_**3(high)**_	1.11	0.96–1.29
**Number of pregnant women**		
**T**_**1(low)**_	1.00	
**T**_**2(intermediate)**_	1.24	1.12–1.36
**T**_**3(high)**_	1.33	1.16–1.51
**Children younger than 4 months exclusively breastfed**		
**T**_**1(low)**_	1.00	
**T**_**2(intermediate)**_	1.21	1.09–1.34
**T**_**3(high)**_	1.35	1.17–1.55
**Children younger than 1 year with up-to-date vaccinations**		
**T**_**1(low)**_	1.00	
**T**_**2(intermediate)**_	1.19	1.08–1.31
**T**_**3(high)**_	1.26	1.10–1.43
**Children aged 12–23 months with up-to-date vaccinations**		
**T**_**1(low)**_	1.00	
**T**_**2(intermediate)**_	1.18	1.07–1.30
**T**_**3(high)**_	1.20	1.05–1.36
**Number of maternal deaths during a given period and place of residence**		
**T**_**1(low)**_	1.00	
**T**_**2(intermediate)**_	0.87	0.52–1.44
**T**_**3(high)**_	1.02	0.49–2.13
**Number of new congenital syphilis cases in children younger than 1 year**		
**T**_**1(low)**_	1.00	
**T**_**2(intermediate)**_	1.50	1.14–1.97
**T**_**3(high)**_	1.29	0.88–1.88

Data on the selected and assessed indicators related to PHC were collected from public-domain sources such as the SIAB local healthcare management tool and the Brazilian Institute of Geography and Statistics [14].

Negative binomial regression models with fixed effects were used for the 79 municipalities in the state of Mato Grosso do Sul, with repeated observations for the selected years (2009–2015). Adjusting the binomial model for longitudinal data allowed us to control for the characteristics of the each municipality that remained constant during the study period, and also permitted estimation of the average strength of an association over the years analyzed for an indicator (response variable). The different indicators we selected were analyzed according to the degree of effect and relative risk (RR), which is easy to interpret when the magnitude of the effect is measured (values above the unit indicate improvements in PHC after the professionals concluded the course and returned to the municipalities where they work). The analyses were conducted with Stata v.14 software (College Station, TX, USA).

## Results

As shown in Table 1, the presence of professionals who completed the specialization course had an effect on some indicators, if the high and intermediate categories (T3 and T2) are considered in comparison to the low reference group (T1). With regard to maternal and child health, intermediate and high increases were seen in enrollment of pregnant women, number of children younger than 4 months exclusively breastfed, children younger than 1 year with up-to-date vaccinations, and children 12–23 months old with up-to-date vaccinations.

Benefits in female preventive healthcare in terms of cervical cancer screening and diagnosis of new congenital syphilis cases in children younger than one year were also observed for T2 (intermediate).

Child development screening programs, number of prenatal care visits, and number of maternal deaths during a given period in a certain place of residence were classified as T1 (low). The other maternal and child health indicators were not presented in the current study because their indices were not relevant.

Table 2 shows the distribution of the mean and standard deviation for professionals who completed the CEABSF specialization course. During 2015, there were an average of 43 graduates per municipality in the state of Mato Grosso do Sul; that same year, each municipality had an average of 229.30 professionals in PHC (including doctors, dentists, nurses, and related professionals). The mean and confidence interval (95% CI) for each indicator can be seen for each year (2009–2015).

**Table 2:** Distribution of course graduates/primary care professionals per municipality and PHC indicators (mean and 95% CI) in Mato Grosso do Sul State (2009–2015).

	2009	2010	2011	2012	2013	2014	2015
**Graduates/municipality**	-	-	2.48 (SD 4.28)	8.69 (SD 6.91)	15.46 (SD 9.06)	21.65 (SD 10.27)	43.80 (SD 36.39)
**Primary care professionals/municipality**	96.92 (SD 255.60)	107.26 (SD 320.36)	169.47 (SD 468.29)	181.80 (SD 494.56)	201.84 (SD 597.82)	217.85 (SD 634.88)	229.70 (SD 706.31)
**Child development screening programs**	2152.79 (1107.78–3197.80)	2009.87 (1058.00–2961.73)	1974.46 (976.70–2972.22)	5581.85 (-1682.31–12846.03)	5613.46 (-1941.29–13168.22)	3145.44 (771.27–5519.60)	1761.72 (1013.49–2509.94)
**Prenatal care**	1557.115 (970.05–2144.17)	1536.17 (949.35–2123.00)	1615.09 (1009.84–2220.33)	1666.87 (972.27–2361.47)	1678.11 (952.72–2403.50)	1588.53 (1058.47–2118.59)	1595.06 (1025.42–2164.69)
**Cervical cancer screenings**	1490.06 (876.16–2103.96)	1341.14 (777.99–1904.29)	1482.84 (877.83–2087.85)	2814.79 (189.94–5439.64)	1549.33 (883.75–2214.91)	1146.66 (784.57–1508.74)	1009.58 (749.49–1269.66)
**Number of pregnant women**	1637.98 (762.49–2513.47)	1606.44 (754.03–2458.86)	1644.11 (772.02–2516.20)	3064.48 (142.55–5986.41)	1529.42 (753.73–2305.11)	1274.02 (699.07–1848.98)	975.68 (693.38–1257.97)
**Children younger than 4 months exclusively breastfed**	842.92 (345.02–1340.82)	812.30 (316.47–1308.14)	837.96 (344.33–1331.58)	2256.17 (-582.06–5094.42)	754.24 (333.64–1174.84)	609.15 (286.23–932.07)	497.62 (336.73–658.50)
**Children younger than 1 year with up-to-date vaccinations**	3347.25 (1519.21–5175.29)	3206.32 (1439.39–4973.25)	3239.84 (1466.56–5013.13)	4666.71 (1373.82–7959.61)	2954.10 (1380.00–4528.19)	2387.31 (1175.22–3599.39)	1863.14 (1281.56–2444.72)
**Children 12–23 months old with up-to-date vaccinations**	3505.14 (1618.46–5391.81)	3510.53 (1596.96–5424.11)	3454.92 (1574.12–5335.72)	4911.87 (1556.24–8267.50)	3200.33 (1478.43–4922.23)	2592.09 (1249.69–3934.49)	1957.74 (1357.81–2557.66)
**Number of maternal deaths during a given period and place of residence**	0.46 (0.23–0.68)	0.38 (0.17–0.59)	0.37 (0.15–0.59)	0.37 (0.14–0.59)	0.28 (0.03–0.52)	0.33 (0.17–0.49)	0.38 (0.17–0.58)
**Number of new congenital syphilis cases in children younger than 1 year**	1.61 (0.36–2.86)	1.58 (0.28–2.89)	1.69 (0.31–3.06)	2.55 (0.27–4.83)	3.21 (0.85–5.58)	3.46 (1.16–5.77)	3.04 (1.31–4.76)
**Gini score**	0.52 (0.52–0.53)						
**Human development index**	0.67 (0.67–0.68)						

## Discussion

The results demonstrate that training had a visible impact on the FHS process, with better indicators related to maternal and child health in locations where professionals who completed the specialization course work.

The most significant indices (enrollment of pregnant women, exclusively breastfed children younger than 4 months of age, children younger than 1 year and children aged 12–23 months with up-to-date vaccinations, cervical cancer screenings, and diagnosis of new cases of congenital syphilis in infants under one year of age) were widely discussed in the course, within specific modules connected to children’s and women’s health and other units such as health surveillance and the work process in family health.

The growth in the number of pregnant women enrolled indicates a concern among these trained professionals for initiating prenatal care, which is seen to be important to the quality of care despite interference from other factors such as poor physical infrastructure, limited access to laboratory testing, and other clinical activities that are still fragile and not particularly effective [15].

These high enrollment numbers also suggest growth in capturing and tracking pregnant women, a topic that is emphasized in the course and supported in literature, to the extent that continuing education initiatives related to women’s health (such as strategies for maternal support) drive active search, pregnancy diagnosis, and early prenatal care [16].

Initiatives such as expanding access and improving the quality of prenatal care within the FHS are necessary, formative, and can have positive impacts in reducing maternal and infant morbidity and mortality, which is corroborated by Rede Cegonha (“The Stork Network”), an institution founded in 2011 to structure and organize maternal and child health care at a national level [15,17]. However, the data indicate that enrollment of pregnant women does not seem to have ensured effective care, since the indicator for prenatal care was not significant, which has been proven in assessment within the Program to Improve Access to and Quality in Basic Care [15].

Monitoring weakness can also be seen in the increase in cases of congenital syphilis diagnosed in infants under one year of age (which had an intermediate impact in this study), despite the recommendation in the Rede Cegonha program to utilize rapid syphilis testing in basic health units [17].

Cases of congenital syphilis are growing at an alarming rate in Brazil and around the world [18], and these cases affect the quality of prenatal care offered, as well as outcomes for health in this population [19,20]. This can be seen in an analysis of access to prenatal care in 2016, which found that 81.0% of mothers of children with congenital syphilis received prenatal care, while 13.6% did not and information was not available for 5.5% [21].

The fact that this preventable infection is growing presents an epidemiological challenge to health services, considering the low effectiveness and quality of prenatal care, inability to capture pregnant women in the system and to diagnose the disease, and difficulties treating women and their partners, which requires compliance and the availability of therapeutic options [19]. It is also partially due to the decrease in condom use, resistance among primary healthcare professionals to administering penicillin [22], technical difficulties using the recommended therapeutic regimen [20], as well as a shortage of penicillin in Brazil since 2014 resulting from a lack of the raw materials needed to manufacture this drug [20].

These issues reinforce the need for strategies to train health professionals so they can adequately manage cases at this level of care, and in this sense regard continuing education in health is one of the pillars of the Brazilian Ministry of Health’s Agenda of Strategic Actions to Reduce Congenital Syphilis in order to train workers and share responsibilities in treating and eradicating syphilis [22].

Although prenatal care for individuals as well as group activities (focused on the integral approach and effective participation of women and their families) has been discussed in the CEABSF course, greater emphasis must be placed on the clinical and assistance aspects required to follow the recommended protocols in order to ensure quality of care, since our results indicate no improvement has been made in the indicator related to prenatal care.

Child development screening programs are a powerful strategy in the FHS to monitor child growth and development, with special attention to prevalent childhood diseases, as well as for activities promoting breastfeeding and immunization [23], which in turn significantly reduce the number of hospitalizations for preventable causes. Even so, the data from this study suggest a certain fragility, since no significant results were seen in terms of child development screening programs, a finding also observed in other studies [24,25] after some activities were not completely implemented and in many cases child assessments were incomplete.

Health education for mothers and caregivers of children should be considered so that these stakeholders can understand the importance of regular monitoring of their children, rather than simply receiving information offered by health professionals, in order to bring care activities beyond a simple complaint-response reaction [23]. From this perspective, a priority should be training health care professionals in child development screening programs and related promotional and preventive activities.

Additionally, vaccination results were also important among the indicators for children, since the number of children under 1 year and aged 12–23 months with up-to-date vaccinations was significant in the areas where course graduates work. It is important to see the impact of the CEABSF course on these indicators, since immunization is one of the most efficient and cost-effective public policies for controlling preventable diseases; as such, it is essential for health professionals to receive ongoing training to develop skills that will motivate parents to vaccinate their children and so that the general population will understand the importance of vaccination [26].

The approach to immunization in the course included developing technical competencies related to the vaccination schedule and encouraging dialog with caregivers and family members about the benefits of vaccination in order to provide a safe place for them to share their understandings, questions, fears and anxieties. This approach was also used to address other topics related to maternal-child health such as breastfeeding, which is still less common than expected in Brazil despite growth in this index and undeniable lifelong benefits for breastfed children, as well as reduced rates of infant mortality and morbidity [27].

Only in recent decades have policies to encourage breastfeeding been implemented in basic care in the country; earlier these measures were restricted to the hospital network, but with these changes exclusive breastfeeding can be continued after mother and children are discharged from the hospital [27]. Studies have shown that forming breastfeeding support groups and providing guidance on this topic in the context of basic care significantly encourage long-term breastfeeding, especially exclusive breastfeeding until 6 months of age [28,29]. Other factors such as maternal age, newborn birth weight, and home visits by the team may also influence the prevalence of exclusive breastfeeding [30].

The increased rate of exclusive breastfeeding up to 4 months of age in municipalities where course graduates work leads us to suggest that there is a connection, since this topic is addressed and discussed within the context of integrating education and service to focus on health promotion and preventing diseases and harm, encouraging the use of health education with collective and group activities that intersect with modules related to life cycles. Conducting group activities for pregnant women and children, as a space to exchange experiences and knowledge among themselves and with healthcare professionals, ensures that health teams hear the needs and questions of these participants, helping to strengthen bonds as well as integral and longitudinal care.

From this perspective, the significant number of pap smears conducted where graduates work seems to demonstrate that access to this testing was easier, and also would imply that women assisted by these professionals were able to obtain information about the importance of cervical cancer screening, possibly because of health education activities conducted.

These initiatives are in line with Ministry of Health recommendations related to the prevention and control of cervical cancer, which is the third most common type of cancer affecting women in Brazil [31]. Pap smear coverage was lower than expected (79.4% in 2013), with regional and sociodemographic disparities [32], and is one of the quality of care indicators recommended by the Program to Improve Access to and Quality in Basic Care [33]. However, this indicator must be analyzed in greater detail, since the majority of these screenings are conducted on women who seek out health services, which indicates the need for active search and other strategies to capture working women and those who do not visit the basic healthcare unit on a routine basis.

Technical difficulties related to sample collection still remain, compromising the quality of the material collected and the diagnostic capacity of screening, and the age range of women who undergo this testing is also challenging; most women screened are under age 35, although the risk of cervical cancer increases after this age [33].

The specialization course analyzed in this paper assumed that participants would develop professional skills based on the responsibility to act from a viewpoint that considered the entire lifespan of users, with emphasis on the lines of care and connecting to the healthcare network to optimize the resources available for the effective care. However, despite the importance of this course in boosting indicators of maternal and child health, some overarching structural barriers that weaken the SUS and small-scale impediments that affect the activities of health professionals and the population’s access to health services in the FHS must be examined more carefully; notable among these are occupational stress [34], especially with regard to working conditions [35], inadequate supplies and infrastructure [36], lack of staff, lack of medications, underfunding of programs, and the precarious socioeconomic conditions faced by the population [37,38].

To minimize the effects of confounding factors, in this study we adopted as a parameter a comparison between the indicators analyzed and others unrelated to the scope of practice for FHS professionals, related to traffic accidents (intermediate ratio: RR: 1.07; IC: 0.95–1.22, and high ratio: RR: 1.01; IC: 0.85–1.21), workplace accidents (intermediate ratio: RR 1.13; IC 0.94–1.36 and high ratio: RR 1.12; IC: 0.87–1.43), and cancer deaths (intermediate ratio: RR: 0.96; IC: 0.89–1.02, and high ratio: RR: 1.01; IC: 0.92–1.12). The results for these indicators were not impacted by the presence of course graduates, validating the choice of statistical model for the type and object of study.

Even considering the pioneering aspects of verifying the effectiveness and impact of a specialized course on family health and maternal and child health care, which can help support other domestic and international studies, this study had some limitations inherent to research using secondary data, such as information quality and the possibility of under-recording. Furthermore, the data only pertain to those professionals in some FHS teams (those who completed a specialization course) which have a limited coverage area, and include findings that suggest intervention of other determinants and intersectoral dimensions linked to health service management.

These results need to be corroborated by investigations that reduce or overcome the limitations mentioned above, and assess aspects of service quality using validated instruments for PHC, as the Primary Care Assessment Tool (PCATool). New studies are recommended in other Brazilian states where specialization courses in family health were conducted with support from UNA-SUS in order to assess other continuing education initiatives for PHC.

## Conclusions

The results of this study demonstrate that the presence of CEABSF graduates in FHS teams improved monitoring and indicators of child and maternal health care with regard to breastfeeding and child vaccination, enrollment of pregnant women, cervical cancer screening, and early diagnosis of congenital syphilis in infants under 1 year of age. New healthcare training courses, particularly in relation to prenatal care and children, would be important to improve the quality of care for women and children.

Additionally, the data represent a starting point for future research and reaffirm the importance and effectiveness of policies on training and continuing education from the Brazilian Ministry of Health/UNA-SUS.

## Supporting information

S1 Data (XLSX)
